# MicroRNA Regulation of Human Breast Cancer Stem Cells

**DOI:** 10.3390/jcm5010002

**Published:** 2015-12-25

**Authors:** Yohei Shimono, Junko Mukohyama, Shun-ichi Nakamura, Hironobu Minami

**Affiliations:** 1 Division of Molecular and Cellular Biology, Kobe University Graduate School of Medicine, Kobe 650-0017, Japan; junkom@med.kobe-u.ac.jp (J.M.); snakamur@kobe-u.ac.jp (S.N.); 2 Division of Biochemistry, Kobe University Graduate School of Medicine, Kobe 650-0017, Japan; 3 Division of Medical Oncology/Hematology, Kobe University Graduate School of Medicine, Kobe 650-0017, Japan; hminami@med.kobe-u.ac.jp

**Keywords:** cancer stem cells, microRNA, Bmi1, Wnt signaling, epithelial-to-mesenchymal transition (EMT), metastasis

## Abstract

MicroRNAs (miRNAs) are involved in virtually all biological processes, including stem cell maintenance, differentiation, and development. The dysregulation of miRNAs is associated with many human diseases including cancer. We have identified a set of miRNAs differentially expressed between human breast cancer stem cells (CSCs) and non-tumorigenic cancer cells. In addition, these miRNAs are similarly upregulated or downregulated in normal mammary stem/progenitor cells. In this review, we mainly describe the miRNAs that are dysregulated in human breast CSCs directly isolated from clinical specimens. The miRNAs and their clusters, such as the miR-200 clusters, miR-183 cluster, miR-221-222 cluster, let-7, miR-142 and miR-214, target the genes and pathways important for stem cell maintenance, such as the self-renewal gene BMI1, apoptosis, Wnt signaling, Notch signaling, and epithelial-to-mesenchymal transition. In addition, the current evidence shows that metastatic breast CSCs acquire a phenotype that is different from the CSCs in a primary site. Thus, clarifying the miRNA regulation of the metastatic breast CSCs will further advance our understanding of the roles of human breast CSCs in tumor progression.

## 1. Breast Cancer Stem Cells

The heterogeneity of tumor cells and the presence of cancer cells with stem cell properties have long been appreciated. For example, the histological resemblance of the tissue of teratocarcinoma to that of the developing fetus was suggested by Virchow in the mid-1800s. The hypothesis that cancers arise from “embryonic rests”, cells leftover from embryogenesis, was proposed in 1875 [1,2]. The genetic studies in leukemia patients demonstrated that a primitive leukemia cell can give rise to fully mature non-replicating progeny, showing that not all cancer cells have the ability to form tumors [3,4,5]. Existence of cancer stem cells (CSCs) was prospectively presented first in leukemia in 1997 [6]. However, evidence for the existence of CSCs in solid tumors has been more difficult to obtain because cells within solid tumors are less accessible, and functional assays suitable for detecting and quantifying normal stem cells from many organs have not been developed. The breast CSCs are the first CSCs prospectively identified from human solid tumors [7]. In 2003, the cells that can cause breast cancer in immunodeficient NOD/SCID mice through serial transplantations were identified. These cells are CD44^+^CD24^−/low^ cancer cells and they establish tumors in recipient animals when as few as one hundred cells are transplanted, whereas tens of thousands of cancer cells with a different marker set fail to form tumors. Then the CSCs are prospectively isolated in various cancers including glioblastoma, colon cancers, and prostate cancers [8].

The CSC hypothesis proposes that tumors are complex systems that recapitulate the complexity of organs or tissues during tumor initiation, maintenance and progression. The CSCs are proposed to have the ability to self-renew and generate differentiated progeny in the same way as normal stem cells in the tissue do. As the cells within a solid tumor are heterogeneous, the cells with CSC properties are still composed of heterogeneous cells. To date, many terms have been used instead of using CSCs, such as tumor-initiating cells and stem-like cells, because of the controversy on the CSC hypothesis or when analyzing the cells whose stem cell abilities are not fully confirmed.

The term CSCs led to some confusion. CSCs do not necessarily arise from normal stem cells by mutations of genes that make the stem cells cancerous [9]. CSCs capable of forming a tumor at one point in time might change during the progression of the disease [9,10]. Genetic and epigenetic factors, such as the acquisition of gene mutations and/or induction of epithelial-to-mesenchymal transition (EMT), affect the generation of CSCs during tumor progression. For example, during the progression to blast crisis in chronic myelogenous leukemia, additional events, including the activation of β-catenin, occur in the granulocyte–macrophage progenitor population, allowing the progenitor cells, not hematopoietic stem cells (HSCs), to become leukemic stem cells [11]. In contrast, in chronic lymphocytic leukemia, the HSCs are involved in leukemogenesis by acquiring the propensity to generate clonal B cells [12]. Thus, the CSCs within an individual tumor may constitute a moving target and the cells that drive growth at one point in time may not be identical to those during tumor progression.

Several experimental methods to characterize CSCs are proposed, including sphere culture, side-population, ALDH1 activity (ALDFLUOR), and limiting dilution assays. Among them, the limiting dilution xenotransplantation assay is considered to be a gold standard to characterize and identify CSCs within a tumor. Because the CSC populations identified by each method contain heterogeneous cancer cells with variable self-renewal and tumorigenic abilities, there will be a little overlap between the CSCs isolated by different purification methods [13]. In addition, it is reported that the gene expression profile changes when the cancer cells in the primary cancer specimens are cultured *in vitro* and/or passaged by xenotransplantation [14]. Breast cancer tissue contains heterogeneous cancer cell populations and clonal selection is observed during the passages by xenotransplantation [15]. Considering that the growth and maintenance of breast CSCs depend on their microenvironment, it is reasonable to speculate that the presence or absence of niche cells, their species differences, and difference of CSC culture methods affect properties and gene expression profiles of breast CSCs. In this review, we mainly focus on the miRNAs specifically expressed in the human breast CSCs directly isolated from the surgical specimens of human breast cancer patients. These miRNAs will help to delineate the molecular regulation of human breast CSCs in breast cancer patients. 

## 2. Shared Properties between Breast CSCs and Normal Mammary Stem/Progenitor Cells

Human tissues maintain their architecture over time through a tightly regulated process of renovation. Under physiological conditions, this process is sustained by a minority of tissue stem cells. The mammary gland develops from a thickening in the ventral skin during embryogenesis that grows into a rudimentary ductal tree by birth [16,17,18]. Then, ductal morphogenesis of the mammary gland occurs largely in the early pubertal period. Pregnancy enhances elongation and side branching of ducts and induces alveologenesis with lactational differentiation. In the murine mammary tissue, *in vitro* and *in vivo* clonality and implantation studies showed that even a single cell within the mammary repopulating unit (MRU) population is able to regenerate whole epithelial tissues of the mammary gland, showing that MRU cells are responsible for the development and maintenance of mammary tissues [19,20].

The mammary epithelium is composed of the inner luminal cell and outer myoepithelium cell layers. The lineage tracing experiments in the mouse identified the luminal and myoepithelial stem/progenitor cells in each layer of the mammary epithelium [21,22]. Thus, it is possible that distinct stem/progenitor cells are responsible for the initial development, homeostasis, and remodeling of the mammary epithelium. In the human mammary epithelium, putative mammary epithelial progenitors have been identified using clonogenicity assays and transplantation assays [23].

Breast CSCs and normal mammary stem cells share a part of the genetic and epigenetic properties that are associated with the regulation of tissue stem cells. We identified that the profile of a set of the 37 miRNAs is shared between human breast CSCs and the stem/progenitor cells of human or murine normal mammary tissues [24]. The findings that transcriptional regulation by SLUG and SOX9 works in both human breast CSCs and normal mammary stem/progenitor cells further show the part of genetic programs shared between these cells [25].

Origin of breast CSCs will differ depending on the tumor subtypes [26]. Comprehensive gene expression profiling revealed the five major molecular subtypes of breast cancer: basal-like, luminal A, luminal B, HER2+/ER−, and normal breast-like. It is generally considered that the luminal compartment or its reprogrammed equivalent will provide breast CSCs. On the other hand, breast CSCs with the gene expression profile similar to basal stem cells exist across the different molecular subtypes of breast cancer [13], suggesting that human breast CSCs use the genetic program for the maintenance of basal stem cells irrespective of tumor subtypes. Understanding the similarity and difference of stem cell properties between human breast CSCs and normal mammary stem/progenitor cells will clarify the roles of CSCs in human breast cancer development and progression.

## 3. miRNAs Specific for Breast CSCs

miRNAs are non-coding RNAs with less than 25 nucleotides and base pairs to their target mRNAs to suppress their translation and/or accelerate degradation. Most miRNAs are evolutionally conserved and have been implicated in a wide variety of cellular processes in both invertebrates and vertebrates [27]. miRNAs are located within intergenic regions, or introns of pre-mRNAs or non-coding RNAs [28,29]. Intergenic miRNAs are located in the regions that are distant from previously annotated genes and constitute an independent transcription unit with a promoter of their own. A minority of miRNAs are derived from the introns of pre-mRNAs or encoded within non-coding RNA genes, suggesting that these miRNAs are dependent on the promoter region of the associated gene and their RNA splicing mechanisms.

miRNAs typically function by base pairing with the 3′ untranslated regions (3′-UTRs) of their target mRNAs through the seed sequences of the miRNAs. The base pairing between a miRNA and its target mRNAs can result in translational inhibition, mRNA destabilization and/or degradation. In this way, miRNAs function as a switch and a fine-tuner of the gene regulatory network [30]. It is also shown that miRNAs can interact with other non-coding RNAs. Competing endogenous RNAs (ceRNAs) are the non-coding RNAs that have multiple binding sites for miRNAs. The interactions of miRNAs with ceRNAs play important roles in the regulation of gene expression, including oncogenes and tumor suppressor genes [31].

miRNAs are especially attractive candidates for regulating stem cell self-renewal and cell fate decisions, as their ability to simultaneously regulate many targets provides a means for coordinated control of concerned gene action [32]. The first two miRNAs to be identified, the *lin-4* and *let-7* in nematode *C. elegans*, control cell divisions in the hypodermal blast lineage and serve as a developmental switch during normal temporal regulation of post-embryonic developmental events [33,34,35]. *lin-4*, the first miRNA identified, mediates translational repression of its target mRNA lin-14, which facilitates the switching of lava of *C. elegans* from stage L1 to L2 and then to L3 [34]. In the absence of either gene, this stem cell lineage fails to differentiate and continues its proliferative cycle. *let-7* is involved in the regulation of the timing of the developmental switch from larval to adult cell fates during *C. elegans* development [35]. Embryonic stem (ES) cell-specific miRNAs, such as miR-302 family, miR-371 cluster, and miR-290 cluster miRNAs, regulate the maintenance and differentiation of ES cells [36,37,38,39].

Cancer cells within a tumor are heterogeneous and miRNAs are differentially expressed between CSCs and other non-tumorigenic cancer cells. Because CSCs are in the minority of cell population in human breast cancers (usually less than 10%), analyses of bulk tumor are unable to identify the miRNAs involved in the regulation of breast CSCs. We isolated breast CSCs directly from the surgically resected specimens of human breast cancer patients and identified a set of miRNAs that are differentially expressed between CSCs and the remaining non-tumorigenic cancer cells [24]. Among them, eight miRNAs are selectively downregulated in the human breast CSC population of 11 human breast cancers. These eight miRNAs are located on the three miRNA clusters and two of the three clusters are the miR-200 clusters, suggesting that the suppression of miR-200 family miRNAs is critically important in the maintenance of stem cell functions.

In addition, other miRNAs, including let-7, miR-1 and miR-27, are among the miRNAs that are differentially expressed between breast CSCs and non-tumorigenic cancer cells ([24,40,41,42,43], for review [8,44,45]). Let-7 family miRNAs are undetectable in ES cells and upregulated upon differentiation [46]. Microprocessor-mediated cleavage and maturation of pri-let-7 miRNAs are blocked by lin-28, a conserved RNA-binding protein and an oncogene [47]. In human breast CSCs, let-7 targets H-RAS and HMGA2 and suppresses self-renewal and differentiation [40]. miR-1 targets the Wnt signaling and suppresses proliferation and migration of breast CSCs [41]. The expression of miR-27 is upregulated by VEGF in breast CSCs and promotes angiogenesis and metastasis [43]. Furthermore, miR-27 targets ectonucleotide pyrophosphatase/phosphodiesterase family member 1 (ENPP1) and regulates the tumorigenicity and drug resistance of breast cancer cells [42]. Downregulation of miR-200 family miRNAs and let-7 family miRNAs is observed in the CSCs of other cancers, such as colon cancer and Wilms tumor, and is associated with cancer progression [48,49]. These evidences suggest that breast CSC-specific miRNAs play important roles in the regulation of self-renewal ability, tumorigenicity, and metastasis.

### 3.1. miR-200 Clusters

Some miRNA genes are clustered in the genome and transcribed as a multi-cistronic primary transcript [50]. Usually, there are between two to three miRNA genes in a cluster. However, larger clusters are also identified, such as the human miR-17-92 and miR-106a-363 clusters, and both of them are composed of six members, which are also conserved in other mammals. It is predicted that a total of 15%–35% of known and predicted miRNA genes in nine selected species constitute clusters under the inter-miRNA distances ranging from 1 kb to 50 kb [51]. miRNAs within a cluster are often, but not always, paralogous with high sequence homology, indicating that they may be the result of genomic duplications.

The polycistronic miR-17-92 cluster is the first microRNA cluster shown to play a role in tumorigenesis [52]. It has two other paralogs in the human genome, the miR-106b-25 cluster and the miR-106a-363 cluster. miR-17-92 and miR-106b-25 are expressed abundantly in a wide spectrum of tissues, but miR-106a-363 is expressed at lower levels. The bicistronic miR-143-145 cluster functions as a tumor suppressor and regulates vascular smooth-muscle cells and mesenchymal cells in the intestine [53,54].

**Figure 1:**
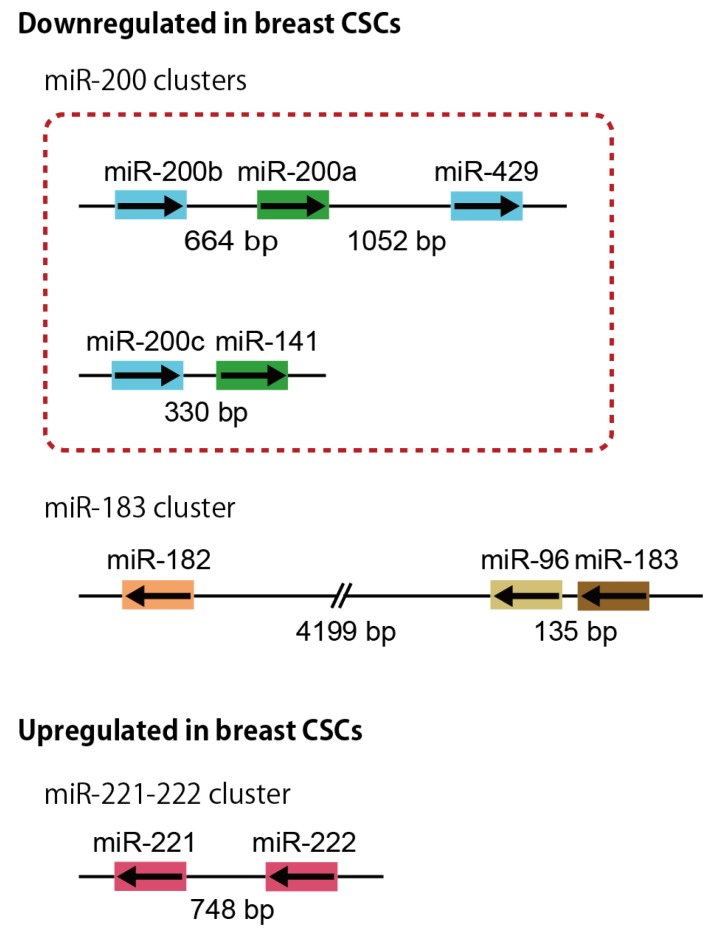
A schematic representation of the miRNA clusters dysregulated in human breast CSCs. The miRNAs sharing the same seed sequence (nucleotides from two to seven) are marked by the same color. The mammalian miR-200 clusters are expressed as two separate polycistronic pri-miRNA transcripts. The miRNAs coded in the miR-200b-200a-429, miR-200c-141 and miR-183-96-182 clusters are downregulated, and those in the miR-221-222 cluster are upregulated in the human breast CSCs. The arrows indicate the direction of the pri-miRNA transcription.

The miR-200 cluster is an extensively studied tumor-suppressive miRNA cluster in the genome (Figure 1). The miR-200 family miRNAs have been highly conserved in deuterostome from Echinodermata and Chordata to all Vertebrata classes, including fish, amphibians, reptiles, birds and mammals [55]. The miR-200 family in mammals is composed of five miRNAs: miR-200a, miR-200b, miR-200c, miR-141, and miR-429. Among them, miR-200a, miR-200b and miR-429 are found in all deuterostomes including Echinodermata, Chordata and Vertebrata, but miR-200c and miR-141 are only detected in cephalochordates, teleosts and mammals or in tunicates, teleosts and mammals, respectively. Invertebrate species such as the fruit fly *D. melanogaster* possess only one orthologue of this family, miR-8 [55,56].

The mammalian miR-200 family clusters are expressed as two separate polycistronic pri-miRNA transcripts: miR-200b-200a-429 and miR-200c-141 clusters (Figure 1). The tricistronic miR-200b-200a-429 cluster is located on mouse chromosome 4 and human chromosome 1p36, whose length of transcript is 6464 bp [57]. The bicistronic miR-200c-141 cluster is located on mouse chromosome 6 and human chromosome 12p13, whose length of transcript is 1211 bp [57]. The miR-200 family members can also be divided into two subgroups based upon their seed sequences that differ by only 1 nt between the subgroups: miR-200b, -200c, and -429 (AAUACUG) and miR-200a and -141 (AACACUG). Furthermore, miR-200 family members belonging to either seed sequence subgroup do not show a clear phylogenetic relationship, suggesting that the 1 nt difference between the two subgroups arose independently in different lineages [56].

The expression of the miR-200 family members can be regulated through interactions with transcriptional factors, modifications of their promoter regions, and Polycomb-group-gene-mediated repression. The promoter regions of the miR-200 family are bound by multiple transcription factors, including zinc finger E-box binding homeobox 1 (ZEB1) and 2 (ZEB2, also known as SIP1), specificity protein 1 (Sp1), Smad3, Wnt inhibitory factor 1 (WIF1) and p53. ZEB1 and ZEB2 can inhibit the transcription of the entire miR-200 family [58]. Sp1 activates the transcription of the miR-200b-200a-429 [59]. p53, Smad3, and WIF1 activate the transcription of miR-200c-141 [60,61,62,63]. The modifications to the promoter regions of each of the miR-200 clusters cause the loss of the expression of the miR-200 family miRNAs in cancer. The miR-200c-141 cluster is silenced by promoter hypermethylation, whereas the miR-200b-200a-429 cluster is silenced primarily through Polycomb-group–mediated histone modifications [64]. miR-22 targets the methylcytosine dioxygenase TET (ten-eleven translocation) family members, inhibits the demethylation of the miR-200 promoter, and suppresses the expression of miR-200 [65].

The mammalian miR-200 family gained particular prominence because it is involved in the regulation of EMT, EGF signaling, regulation of stem cell characters, and somatic cell reprogramming into induced pluripotent stem cells [24,56,66,67,68,69,70,71,72]. EGF signaling induces EMT, and EGF is also targeted by miR-96 which is downregulated in breast CSCs [73]. A large number of studies demonstrate the strong suppressive effects of miR-200 on cell transformation, cancer cell proliferation, migration, invasion, tumor growth and metastasis [61]. The roles of miR-200 family miRNAs in breast CSCs are described in more detail later in Section 4.

### 3.2. miR-183 Cluster

The miR-183 cluster, which is comprised of miRNA-183, -96 and -182, is a miRNA family with sequence homology (Figure 1). The tricistronic miR-183 cluster is located on mouse chromosome 6 and human chromosome 7, whose length of transcript is 19121 bp [57]. Despite the strong similarity in the sequences of these miRNAs, minute differences in their seed sequences result in both overlapping and distinct targets, which are often within the same pathway. These miRNAs have tightly synchronized expression during development and are required for maturation of sensory organs [74]. The miR-183 cluster is highly and widely conserved in protostomes and deuterostomes [74,75]. Although the chromosomal order of miR-183, -96, -182 is conserved in deuterostomes, their location and the intergenic spacing between the miRNA genes vary between species.

The miR-183 cluster miRNAs are frequently upregulated in a variety of non-sensory diseases, including cancer. However, the miR-183 cluster miRNAs are downregulated in the human breast CSCs, suggesting that suppression of the miR-183 cluster is required for the maintenance of CSC properties. The fact that common targets of miR-183 cluster miRNAs include SNAI2, SMAD4, β-catenin and Bmi1 suggests that the downregulation of miR-183 cluster in breast CSCs is associated with activation of EMT, self-renewal and Wnt signaling [67,74,76]. The transcription of the miR-183 cluster is upregulated by transcription factors, such as β-catenin/TCF/LEF and TGF-β, and is downregulated by GATA-3, ZEB1 and DNA methylation [76,77,78,79]. There are several CpG islands before the miR-183 transcription start site (3.5, 8 and 10 kb) that are epigenetically regulated by DNA methylation. In addition, secondary transcription start sites are identified, suggesting that the independent regulation for each miRNA exists in the miR-183 cluster.

### 3.3. miR-221-222 Cluster

The miR-222-221 cluster, which is composed of miR-221 and miR-222, is located in tandem on human chromosome Xp11 and is transcribed as a single RNA precursor with RNA polymerase II [80]. The expression of the miR-221-222 cluster is upregulated by angiotensin II, HMGB1, NF-ĸB, HOXB7/pBX2, and microphthalmia-associated transcription factor (MITF), and is downregulated by promyelocytic leukemia zinc finger (PLZF) and a repressive complex formed by estrogen receptor α (ERα) and two nuclear receptor corepressors, NCOR1 and NCOR2 [80,81,82,83]. The miR-222-221 cluster is highly conserved in Vertebrata classes, including mouse, rat and human [84].

miR-221 and miR-222 have the same seed sequence, and they mostly function as oncogenes in human epithelial tumors [84,85]. They also function as tumor suppressors in some tumors, such as erythroleukemia [86]. miR-221 and miR-222 regulate cell cycle progression, apoptosis, cell migration and stemness by targeting cell cycle inhibitors CDKN1B (p27^Kip1^) and CDKN1C(p57^Kip2^), PUMA, FOXO3, PTEN, Bim, c-Kit, TIMP3, ER-α and DNA methyltransferase DNMT3b [82,84,87].

The miR-222-221 cluster miRNAs are upregulated in the human breast CSCs and normal mammary stem/progenitor cells (Figure 1) [24]. Upregulation of miR-221 and/or miR-222 is observed in CSCs isolated from pancreas and glioblastoma cancer cells [88,89]. miR-221 is involved in the promotion of an aggressive basal-like phenotype in breast cancer, functions downstream of the RAS pathway and triggers EMT [90,91].

### 3.4. miR-142

miR-142 is broadly expressed in various hematopoietic lineages, and plays important functions in hematopoiesis, immune responses, and T cell differentiation [92,93,94,95]. miR-142 is located at a genomic locus associated with t(8;17) translocation in B-cell leukemia and is mutated in diffuse large B-cell lymphoma [96]. In contrast, miR-142 is expressed at low levels in many other cell types. Consistent with these findings, miR-142-null mice and miR-142 gene trap mice show the impairment of hematopoietic lineage formation [97,98]. The transcription start site of miR-142 is located 1205 base pairs upstream of the precursor sequence within a highly conserved CpG island and the transcription of the pri-miR-142 is epigenetically repressed by DNA methylation [99]. In addition, a second CpG island overlapped with the precursor.

miR-142 is very highly expressed in a human breast CSC population, but is undetectable in a normal mammary stem cell population [24,100]. We and others show that miR-142 targets APC and activates the canonical Wnt signaling pathway and further activates the transcription of miR-150 which is also upregulated in human breast CSCs. The regulation of the Wnt signaling pathway by miR-142 and other miRNAs is described in more detail later in Section 4.3.

### 3.5. miR-214

miR-214, together with miR-199a-2, is located inside the sequence of the long non-coding Dynamin 3 opposite strand (Dnm3os) transcript on mouse and human chromosome 1. miR-214 is upregulated or downregulated in human tumors [101]. miR-214 is one of the miRNAs highly upregulated in human breast CSCs. And the miR-214 locus is frequently amplified in breast cancers [102]. miR-214 is upregulated in luminal A, normal-like and triple-negative subtypes, but it is not upregulated in other subtypes [101,103,104]. Ubiquitous miR-214-specific knockout mice are viable and fertile, but following ischemia-reperfusion injury, show impaired cardiac function and progression to heart failure [105]. In contrast, mice lacking Dnm3os, which encodes miR-214 and miR-199a-2, display severe skeletal defects and die within the first month of birth [106]. Further studies are required to clarify the roles of miR-214, miR-199a-2, and long non-coding Dnm3os in development.

miR-214 regulates cell differentiation, stemness, apoptosis, and invasion by targeting Ezh2, p53, transcription factor TFAP2, PTEN, BIM, and β-catenin [107,108,109,110]. In ovarian cancer, miR-214 increases CSCs by upregulating the Nanog expression [107]. miR-214 induces cell survival by targeting PTEN and BIM [108]. miR-214 induces cell invasion by targeting p53 [109]. In contrast, miR-214 suppresses stem-like traits in human hepatocellular carcinoma cells by directly targeting Ezh2 and β-catenin [110], suggesting that the roles of miR-214 will be different depending on the tumor types. Thus, miR-214 seems to have important roles in the regulation of proliferation, differentiation, stemness, apoptosis, invasion and metastasis [101].

## 4. Signaling Pathways and Genes Targeted by miRNAs Specific to the Breast CSCs

Multiple miRNAs and miRNA clusters specifically dysregulated in human breast CSCs coordinately target the signaling pathways and genes that have important roles in the maintenance and regulation of breast CSCs and normal mammary stem/progenitor cells. These signaling pathways and genes include a self-renewal factor Bmi-1, the apoptosis signaling pathway, the canonical Wnt signaling pathway, the Notch signaling pathway, and EMT.

### 4.1. Self-Renewal Factor Bmi1

Many tissues are maintained throughout the lifespan of an organism by a small number of adult stem cells. These cells are unique in that they have both the ability to give rise to new stem cells via self-renewal and the ability to differentiate into the mature cells of a tissue. To maintain tissue homeostasis, stem cells have developed strict regulatory mechanisms to self-renew, differentiate, and prevent premature senescence and apoptosis.

**Figure 2:**
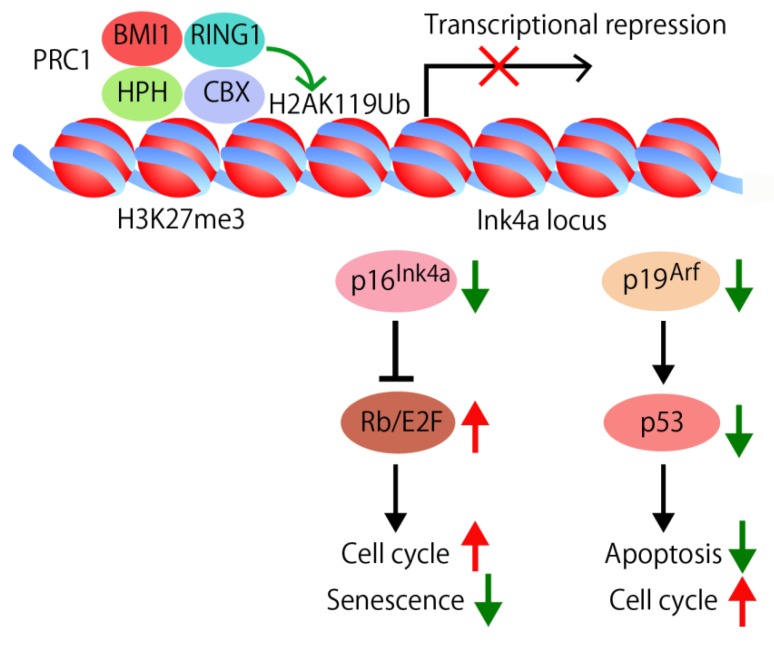
Regulation of cell cycle, apoptosis, and senescence by self-renewal factor Bmi1. Bmi1, a component of PRC1, is involved in the stem cell maintenance in multiple tissues and organs. PRC1 suppresses the Ink4a locus that encodes the *p16^Ink4a^* and the *p19^Arf^* genes through the specific biochemical histone modifications, such as the trimethylation of the H3-K27 (H3K27me3) and the ubiquitination of H2A-K119 (H2AK119Ub). The chromodomain of CBX binds to H3K27me3 and RING1 deposits monoubiquitin on H2AK119. In the absence of p16^Ink4a^, the cyclin D/Cdk4/6 complex can phosphorylate RB, allowing the E2F-dependent transcription which leads to cell cycle progression. In the absence of p19^Arf^, MDM2-mediated p53 degradation causes low p53 levels, thus preventing cell cycle arrest and apoptosis. In addition, the gradual accumulation of p16^Ink4a^ expression during physiological aging implicates that p16^Ink4a^ is involved in the regulation of senescence.

Bmi1 is a component of Polycomb repressive complex 1 (PRC1). Analyses of Bmi1 knockout mice showed that Bmi1 is involved in the stem cell maintenance in multiple tissues and organs, including hematopoiesis, skeletal patterning, neurological functions, and development of the cerebellum [111]. Bmi1 is also essential for the self-renewal of multiple tissues and organs including mammary tissues [112]. To support self-renewal of somatic stem cells, PRC1 suppresses the expression of the Ink4a locus that encodes the *p16^Ink4a^* and the *p19^Arf^* genes and other genomic loci through specific biochemical histone modifications, the addition of trimethyl groups to the H3-K27 amino acid residue and of ubiquitin protein to H2A-K119 (Figure 2). The deubiquitinating enzyme USP16 removes the ubiquitin protein from H2A-K119, and upregulates the transcription of the Ink4a locus [113]. In this way, USP16 antagonizes the self-renewal and senescence pathways in multiple tissues.

Bmi1 was initially identified as an oncogene cooperating with c-myc in a murine model of lymphoma ([114,115], for review [116]). Bmi1 suppresses the *p16^Ink4a^* and the *p19^Arf^* genes coded in the Ink4a locus and regulates senescence, cell cycle, and apoptosis (Figure 2) [116,117]. In the absence of Bmi1, p16^Ink4a^ is upregulated and prevents binding of Cdk4/6 to cyclin D. The inhibition of the kinase activity of Cdk4/6 results in hypophosphorylation of pRB, leading to cell cycle arrest and senescence [117]. The gradual accumulation of p16^Ink4a^ expression during physiological aging and several aging-associated diseases directly implicates that p16^Ink4a^ is involved in the aging process [118]. p19^Arf^ is another target suppressed by Bmi1 (Figure 2). p19^Arf^ sequesters mouse double minute 2 (MDM2) and inhibits p53 degradation. In the absence of Bmi1, p19^Arf^ and p53 are upregulated, resulting in p53-mediated cell cycle arrest and apoptosis [119,120]. Point mutations and deletion of *p16^Ink4a^* and *p19^Arf^* are frequently found in many types of human cancers, which implicate them as key regulators of immortalization and/or senescence checkpoints. The observation that Bmi1 is essential for the self-renewal of multiple adult tissues and organs in part via repression of the genes involved in senescence, apoptosis, and cell cycle progression suggests that stem cells have evolved specific mechanisms to repress senescence and to prolong their capacity to proliferate [116].

**Figure 3:**
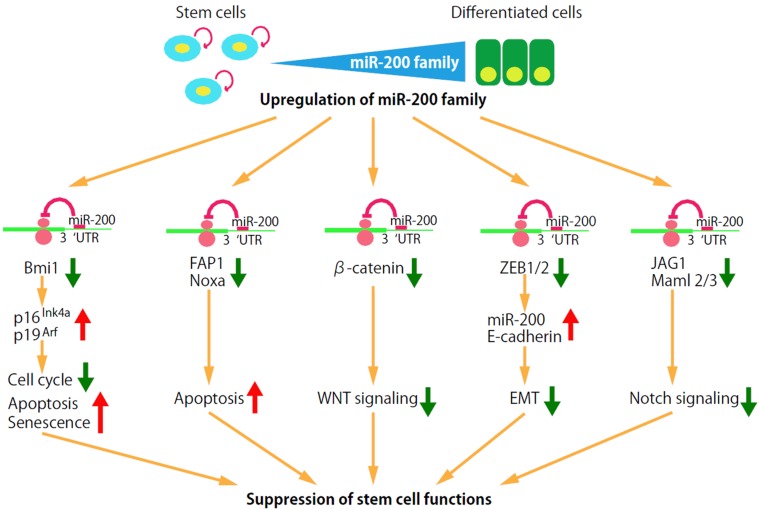
Targeting of the genes and pathways for stem cell maintenance by miR-200 family miRNAs. Expression of the miR-200 family miRNAs is downregulated in the breast CSCs and normal mammary stem/progenitor cells, and is upregulated in the more differentiated counterparts. The miR-200 family miRNAs are involved in the regulation stem cell functions by targeting the genes and pathways important for stem cell maintenance, such as self-renewal factor Bmi-1, the apoptosis signaling pathway, the canonical Wnt signaling pathway, EMT and the Notch signaling pathway.

The expression of Bmi1 is regulated by miRNAs, such as miR-128, miR-200b/c, miR-141, miR-15, miR-16, miR-203, miR-183, miR-194, and miR-218 [24,67,121,122,123,124,125]. Among them, miR-200b, miR-200c, miR-141, and miR-183 are specifically downregulated in the human breast CSCs and normal mammary stem/progenitor cells [24], suggesting that miRNAs are important regulators of self-renewal abilities in breast CSCs and normal mammary stem/progenitor cells (Figure 3). miR-200c strongly suppresses the ability of human breast CSCs to form tumor when engrafted into the mammary fat pad region of immunodeficient mice [24]. miR-200c also suppresses the ability of normal mammary stem cells to form mammary ducts when engrafted into the mammary fat pad of the syngeneic mice. These findings suggest that the three miRNA clusters, namely two miR-200 clusters and one miR-183 cluster, coordinately upregulate the expression of Bmi1 to enhance the stem cell self-renewal abilities in both breast CSCs and normal mammary stem/progenitor cells.

### 4.2. Apoptosis Signaling Pathway

miR-200b, miR-200c and miR-141 target Bmi1 which suppresses p53-mediated cell cycle arrest and apoptosis by repressing p19^Arf^(Figure 2 and Figure 3). In addition, miR-200c functions as an enhancer of apoptosis by targeting molecules such as FAP-1, a known inhibitor of CD95-mediated apoptosis, and Noxa, a member of the Bcl-2 family (Figure 3) [126,127]. The miR-200b, -200c and -429 subgroup miRNAs, but not the miR-200a and -141 subgroup miRNAs, target PLCγ1, reduce cell viability and induce apoptosis [71].

The miR-221-222 miRNA cluster is upregulated in the human breast CSCs and normal mammary stem/progenitor cells. miR-221 and miR-222 induce cell cycle progression and suppression of apoptosis by targeting cell cycle inhibitors p27 and p57, and Bcl-2 homology 3 (BH3)-only Bcl-2 family member PUMA [128,129,130]. These findings suggest that dysregulation of the three miRNA clusters, namely the downregulation of the miR-200 clusters and the upregulation of the miR-221-222 cluster, is involved in the suppression of apoptosis in both breast CSCs and normal mammary stem/progenitor cells.

### 4.3. Wnt Signaling

The fact that some cancer cells share the extended self-renewal ability with normal stem cells and that the canonical Wnt signaling pathway is implicated in both stem cell self-renewal and cancer suggest that the normal physiological regulator of stem cell functions might be “hijacked” in cancer [131]. In 1982, Nusse and Varmus identified the mouse proto-oncogene Wnt1 (Int1) in Wnt signaling [132]. Wnt regulates cell proliferation, differentiation, adhesion, migration and stem cell self-renewal through β-catenin-dependent (canonical) and β-catenin-independent (non-canonical) Wnt signaling pathways [133]. In human colon cancers, mutations in the *Adenomatous polyposis coli* (*APC*) gene are the most common known acquired genetic change for the aberrant activation of the canonical Wnt signaling pathway during tumor development and progression [134,135,136]. APC is a component of the destruction complex that destabilizes β-catenin and suppresses the activity of the canonical Wnt signaling pathway. In a model for the stepwise progression of colon tumorigenesis, *APC* gene mutations play an important role in the initiation step, followed by successive mutations in other genes, including *K-Ras* and *p53* [137].

The expression of APC is not limited to the intestine, but is widely observed in many other tissues, including lung, liver, kidney, and mammary tissue. However, the role of the suppression of APC and the activation of the canonical Wnt signaling in the tumor initiation process of the tissues other than the colon largely remain unknown, because *APC* mutations are less frequent in tumors originating from these tissues. For example, recent data from The Cancer Genome Atlas (TCGA) reveals a ~2% incidence of *APC* mutations in human breast cancer (the TCGA Research Network: http://cancergenome.nih.gov/).

miRNAs target Wnt signaling components and dysregulate the activity of the canonical Wnt signaling. For example, miRNAs, such as miR-135, miR-27, mir-155, miR-129, miR-106b, let-7, miR-125, miR-663, and miR-142, target APC and activate canonical Wnt signaling [100,138,139,140,141,142,143,144,145,146]. miR-29 targets the negative regulators of Wnt signaling, such as Dikkopf-1 (Dkk1), Kremen2, and secreted frizzled related protein 2 (sFRP2) and activates the Wnt signaling pathway [147]. Furthermore, the Wnt signaling pathway is activated by the miRNAs that target other inhibitors of the Wnt signaling pathway [148].

**Figure 4:**
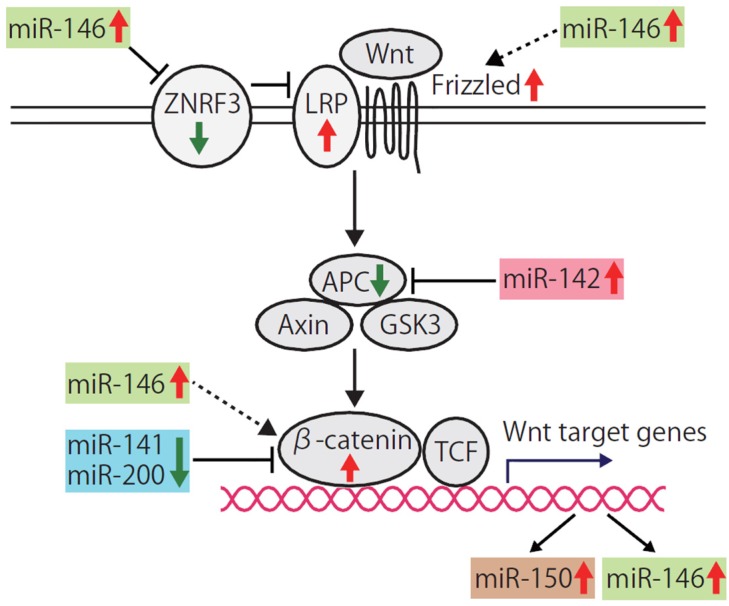
Activation of the canonical Wnt signaling pathway by the breast CSC-specific miRNAs. The canonical Wnt signaling pathway is implicated in both stem cell self-renewal and cancer. The multiple miRNAs dysregulated in the breast CSCs, such as miR-142, miR-146, miR-200, and miR-141, cooperatively activate the Wnt signaling pathway by targeting or upregulating the expression of its components. The activation of the Wnt signaling pathway induces the transcription of the Wnt target genes, including miR-146 and miR-150. miR-150 enhances the proliferation of mammary epithelial cells. Upregulation of miR-146 further enhances the activity of the Wnt signaling pathway in a positive feedback manner.

The profile of a set of the 37 miRNAs is mostly shared between human breast CSCs and the stem/progenitor cells of human or murine normal mammary tissues, but miR-142 is exceptional; miR-142 is very highly expressed in the human breast CSC population, but is undetectable in a normal mammary stem cell population [24,100]. We and others show that miR-142 targets APC and activates the canonical Wnt signaling pathway (Figure 4) [97,100,141,146,148]. Knockdown of miR-142 upregulates the APC expression, reduces the clonogenicity of human breast CSCs *in vitro*, and suppresses the tumor growth initiated by the human breast CSCs *in vivo* [100]. Furthermore, aberrantly proliferating dysplastic mammary tissues are formed when the mammary stem cells overexpressing miR-142 are transplanted into the mammary fat pad of the syngeneic mouse.

These findings propose a novel mechanism for the activation of the Wnt signaling in breast cancers in which the gene mutations involved in the activation of the Wnt signaling pathway are less frequent; the Wnt signaling pathway is epigenetically transactivated by miR-142 in human breast CSCs. Analyses of bulk tumor will be unable to identify the important roles of miR-142 in breast cancer because CSCs are a minority population in human breast cancers (usually less than 10%). Thus, focusing on the CSCs that are a minority population in breast cancer tissue will have a potential to uncover molecular mechanisms that are important for cancer development and progression.

miR-150 is a miRNA specifically expressed in mature lymphocytes and its premature expression blocks B-cell development [149]. We identified that miR-150 is expressed in the mammary epithelium and its expression is higher in breast CSCs and normal mammary stem/progenitor cells [24,100]. The promoter region of miR-150 precursor is targeted by T-cell factor (TCF)/β-catenin and the transcription of miR-150 is activated by miR-142 which activates the canonical Wnt signaling pathway (Figure 4) [100]. Mammary cells overexpressing miR-150 form a hyperplastic mammary tree with extremely increased branching and thick mammary ducts. However, unlike miR-142, miR150 does not induce the dysplastic change of the mammary tissue. A simple model to explain the upregulation of miR-142 and miR-150 in human breast CSCs is that suppression of the APC protein expression by miR-142 increases the activity of the canonical Wnt signaling pathway and thereby enhances miR-150 expression.

miR-200 family miRNAs that are downregulated in human breast CSCs and normal mammary stem/progenitor cells function as the suppressors of the Wnt signaling pathways (Figure 3 and Figure 4). miR-200a and miR-141 suppress the Wnt signaling pathway by targeting β-catenin [150,151]. miR-8, a *Drosophila* homologue of miR-200, targets TCF transcription factor and suppresses the Wnt signaling activities [152].

miR-146 is a miRNA upregulated in human breast CSCs and functions as the enhancer of the Wnt signaling pathways (Figure 4) [24]. miR-146 targets Zinc RING finger 3 (ZNRF3), an E3 ubiquitin ligase and an antagonist of Wnt signaling in papillary thyroid carcinoma cells (Figure 4) [153]. ZNRF3 removes Wnt receptors from the stem cell surface [154]. In addition, cell membrane levels of FZD6 and LRP6 are increased by miR-146, which further activate the Wnt/β-catenin signaling [153]. Furthermore, miR-146a maintains the Wnt signaling activity by stabilizing β-catenin and induces symmetrical cell division of CSCs (Figure 4) [155]. Because the expression of miR-146a is induced by Snail and β-catenin, the Snail-miR-146a-β-catenin loop plays an important role in the maintenance of the activity of the Wnt signaling pathway. These findings suggest that the multiple miRNAs specific to the breast CSCs coordinately upregulate the Wnt signaling pathway in both breast CSCs and normal mammary stem/progenitor cells.

### 4.4. EMT

EMT is a process by which a normally polar, epithelial cell undergoes a change to a mesenchymal-like cell. By undergoing EMT, a cell is able to take on the characteristics of a mesenchymal cell and becomes more motile and invasive. In human breast cancer cells, the canonical Wnt signaling induces the expression of intracellular protein Axin2 to stabilize EMT-transcription factor Snail and induces EMT [156]. EMT is linked to the progression of cancer and increases stemness of breast CSCs and mammary stem/progenitor cells [157].

The miR-200 family is highly expressed within epithelial cells and miR-200c and miR-141 have both been strongly linked to epithelial integrity [158,159]. The miR-200 family miRNAs downregulate ZEB1 and ZEB2 expression, and effectively upregulate the cellular E-cadherin level to maintain a cell in a more epithelial-like state (Figure 3). ZEB1 suppresses the expression of all miR-200 family members (miR-141, miR-200a,b,c and miR-429), which in turn inhibits the translation of ZEB1 mRNA, resulting in the double-negative ZEB/miR-200 feedback loop [160]. Thus, ZEB1 and ZEB2 keep a cell in a mesenchymal phenotype by repressing the transcription of both E-cadherin and the miR-200 family miRNAs.

miR-22 targets the ten-eleven-translocation (TET) family of methylcytosine dioxygenases and demethylates the promoter region of the miR-200 precursor [161]. Interestingly, the cooperation between miR-22 and the miR-200 family results in EMT, an elevated pool of stem cells and increased tumorigenesis. Therefore, the interplay between the miR-200 family, miR-22, and ZEB1/ZEB2 plays an important role in the stemness regulation and EMT.

Slug, Snail, and Twist are the transcriptional factors that trigger EMT which is connected to the stem cell phenotype. Although these transcription factors induce EMT, they have distinct roles, especially during development. For example, Slug directly transactivates ZEB1, but Snail works indirectly in this transactivation [162]. Among these transcription factors, Slug cooperates the transcription factor Sox9 in breast cancer cells and normal mammary stem/progenitor cells [25]. Inhibition of either Slug or Sox9 blocks the activities of normal mammary stem/progenitor cells and that of breast cancer cells, suggesting that breast cancer cells and normal stem/progenitor cells are controlled by similar key regulators. Analyses of the genetically engineered knock-in reporter mouse lines confirmed that Slug regulates mammary stem cells [163]. In contrast, Snail serves as the regulator of CSCs of MMTV-PyMT mouse mammary tumor, whose formation appears to be driven primarily from luminal mammary epithelial cells. As discussed in Section 2, breast CSCs with a gene expression profile similar to basal stem cells exist across the different molecular subtypes of breast cancer [13], suggesting that human breast CSCs use the genetic program for the maintenance of basal stem cells irrespective of tumor subtypes. Therefore, understanding the roles of Snail and Slug in mammary basal stem cell-derived tumors is required to further clarify the roles of Snail and Slug in human breast CSCs.

We found that miR-199a, a miRNA upregulated in human breast CSCs, targets Snail and suppresses its expression [164]. And miR-182 targets Slug and induces mesenchymal-to-epithelial transition (MET) features in prostate cells [165]. Because miR-199a is highly upregulated and miR-182 is downregulated in human breast CSCs and mammary stem/progenitor cells, it is possible that miRNAs function as epigenetic regulators of the EMT transcription factors Snail and Slug to regulate the stem cell abilities of breast CSCs and normal mammary stem/progenitor cells.

### 4.5. Notch Signaling Pathway

The Notch signaling pathway is associated with the regulation of cell fate at several distinct developmental stages of the mammary gland and has been implicated in cancer initiation and progression [166,167,168]. In addition to its central role in development, the Notch signaling pathway is deregulated in a number of cancers. Nevertheless, mutations in Notch pathway components are rare in solid tumors.

The activation of the Notch signaling pathway occurs when Notch receptors bind to one of the membrane-bound Notch ligands, such as Jagged1 (JAG1), Jagged2 (JAG2), Delta-like 1 (DLL1), Delta-like 3 (DLL3), and Delta-like 4 (DLL4). Ligand binding causes a conformational change in the Notch receptor and leads to a sequence of proteolytic cleavage events in the receptor. A γ-secretase releases the intracellular domain of Notch (NICD), allowing it to translocate to the nucleus to activate the expression of target genes, including the Hairy/enhancer of split (Hes) family genes, the cell cycle regulator p21 and cyclin D1 [169].

miR-200 family miRNAs suppress the Notch signaling by targeting Notch pathway components, such as JAG1 and the mastermind-like Notch coactivators, Maml2 and Maml3 (Figure 3) [170]. miR-146a, a miRNA upregulated in human breast CSCs, also activates the Notch signaling pathway by targeting Numb, a suppressor of the Notch signaling pathway [171,172]. These findings suggest that the downregulation of miR-200 members and upregulation of miR-146 are involved in the activation of the Notch signaling pathway in the breast CSCs and normal mammary stem/progenitor cells.

## 5. Metastatic CSC Specific miRNAs

Systemic dissemination and metastasis are responsible for most cancer-related deaths. The breast cancer metastases appear years or even decades after the surgical removal of the primary tumor [173]. Current evidence shows that metastases are initiated by metastasis-initiating cells with stem cell abilities. The CSC marker CD44^+^ breast cancer cells in the lung metastases are highly enriched for tumor-initiating abilities [174]. Similarly, CD133^+^/CXCR4^+^ cells, a subfraction of the putative pancreatic CSCs present at the invasive front of cell line-induced pancreatic tumors, are enriched for metastatic capabilities [175]. Along the same lines, CD26, in combination with the CSC marker CD133, has been proposed as a marker for the colorectal metastatic CSC population in primary tumor xenografts [176]. Furthermore, the early stage metastatic cells in the human breast cancer patient-derived xenograft (PDX) mice are characterized by the expression of the stem cell genes, together with EMT, prosurvival and dormant associated genes [177].

The breast tumor growth and metastasis are analyzed in the PDX models of human breast cancer. These tumor grafts illustrate the diversity of human breast cancer and maintain essential features of the original tumors, including metastasis to specific sites. Tumor engraftment into mice is a prognostic indicator of disease outcome for women with newly diagnosed breast cancer [178]. The tumor cells in the lung metastases of the PDX models exhibit the epithelial and differentiated statuses that are different from the cells in the primary site [179]. We found that metastatic cancer cells in the lung of the PDX mice are in the dormant state which is characterized by the reduced expression of cell surface CXCR4 expression [180,181]. Spontaneous metastases observed in these breast cancer PDX models potentially recapitulate the process of metastasis of cancer cells in human breast cancer patients.

Several miRNAs that are associated with metastatic CSCs are identified. miR-33b inhibits the stemness, migration and invasion of metastatic breast cancer cells is by targeting HMGA2, SALL4 and Twist1 [182]. miR-199a suppresses the expression of FOXP2 and promotes breast cancer CSC propagation, tumor initiation, and metastasis [183]. miR-20a downregulates MICA and MICB, two ligands for the stimulatory NK cell receptor NKG2D, in breast CSCs and enhances the metastatic abilities by promoting the resistance of breast CSC to NK cell cytotoxicity [184]. miR-7 inhibites the metastasis of breast CSCs by targeting SETDB1 and reducing the expression of c-myc, twist, and mir-9, the downstream target genes of the STAT3 pathway [185]. It is also shown that lncRNA linc-ROR enhanced breast cancer cell migration, invasion and stem cell properties [186]. linc-ROR is associated with miRNA ribonucleoprotein complexes (miRNPs) and functions as a ceRNA to mi-205, thereby preventing the degradation of mir-205 target genes, including EMT inducer ZEB2.

miRNAs are selectively incorporated in exosomes, membrane vesicles of an average 30–100 nm diameter. Exosomes are formed within the multivesicular bodies (MVBs), also known as late endosomes, and are released upon the fusion of MVBs with the plasma membrane [187]. The mechanism of exosome-mediated cell-to-cell communication is important in the regulation of cell growth and dissemination of cancer cells, since cancer cells constitutively secrete exosomes and can target both locally adjacent cells and cells located at distant organs [188]. For example, miR-181c in exosomes targets PDPK1 which regulates actin-dynamics by regulating the phosphorylation of cofilin, and promotes the destruction of the blood-brain barrier through the abnormal localization of actin [189]. Furthermore, the metastatic mouse mammary tumor 4T1 cells, but not the poorly metastatic mammary tumor 4TO7 cells, can secrete miR-200 family miRNAs into exosomes [190]. The poorly metastatic 4TO7 cells can take up miR-200 from the exosomes of 4T1 cells and become metastatic in a miR-200-dependent manner. This study provided novel evidence showing that metastatic capability can be transferred from metastatic to non-metastatic cancer cells through exosomes. In addition, this finding suggests that circulating miRNAs are not only just cancer biomarkers, but they are also functional, being capable of promoting metastasis *in vivo*.

Human metastatic CSCs will initiate metastatic colonization by adapting to a distant tissue microenvironment. [10]. For example, metastatic breast cancer cells express many osteoblast-related genes (osteomimicry) that promote its metastasis to the bone [191]. miRNAs play an important role in regulating osteoblast differentiation and also function as regulators of bone metastases [192,193]. miR-218 is significantly upregulated during osteoblast differentiation and targets the inhibitors of Wnt signaling [194]. Thus, miR-218 found in metastatic breast cancer cells is a potent activator of Wnt signaling and promotes the osteomimicry to facilitate bone metastasis of breast cancer cells. In addition, miR-200 family miRNAs are involved in colonization and metastases to distant organs [195]. Because the expression of miR-200 family is downregulated in breast CSCs, analyses of the metastatic CSCs will be required to further characterize the roles of miR-200 family miRNAs in colonization and metastases to distant organs. Uncovering the gene and miRNA expression in the metastatic CSCs is required to characterize the metastatic CSCs and find ways to target them.

## 6. Future Perspectives

miRNAs work as a part of the epigenetic program that regulates the stem cell abilities of both breast CSCs and normal mammary stem/progenitor cells. The miRNAs specifically expressed in breast CSCs target the genes and the signaling pathways important for the regulation of stem cell properties of CSCs. In addition, it will become clearer that miRNA regulation is involved in the initiation, dormancy, and establishment of metastases driven by the metastatic CSCs.

Several small RNA-based drugs are under clinical trials. Fomivirsen was the first RNA-based drug approved by the US Food and Drug Administration (FDA) in 1998 [196]. It is a synthetic, modified, 21-long antisense oligonucleotide used as an antiviral for the treatment of cytomegalovirus retinitis. MRX34 (Mirna therapeutics, Inc., Austin, TX, USA) is a liposomal miR-34 mimicis designed to deliver a mimic of the naturally occurring tumor suppressor miR-34 [197]. The most advanced miRNA trial involves the use of anti-miR-122 (miravirsen) for hepatitis C therapy, which shows a reduction in viral RNA with no evidence of resistance [198]. However, miRNA therapeutics are still in their infancy.

The CSC-targeting therapy has the potential to improve the prognosis of breast cancer patients by attaching the CSCs in the primary sites and suppressing recurrence driven by the metastatic CSCs. Considering that the miRNAs are important regulators of CSCs, miRNA therapeutics will be one of the therapeutic interventions for the CSC-targeting therapies that suppress tumor progression and metastasis.
